# Development of a predictive scoring system for vitamin D deficiency ‘Vitamin D Deficiency Predicting Scoring (ViDDPreS)’ based on the vitamin D status in young Japanese women: a nationwide cross-sectional study

**DOI:** 10.1017/S1368980024001708

**Published:** 2024-09-27

**Authors:** Akiko Kuwabara, Eiji Nakatani, Hideaki Nakajima, Satoshi Sasaki, Kenichi Kohno, Kazuhiro Uenishi, Masaru Takenaka, Kyoko Takahashi, Akihiro Maeta, Nobuko Sera, Kaori Kaimoto, Masako Iwamoto, Hisaya Kawate, Mayumi Yoshida, Kiyoshi Tanaka, Naoko Tsugawa

**Affiliations:** 1 Department of Nutrition, Graduate School of Human Life and Ecology, Osaka Metropolitan University, Osaka, Japan; 2 Division of Medical Statistics, Graduate School of Public Health, Shizuoka Graduate University of Public Health, Shizuoka, Japan; 3 Earth System Division, National Institute for Environmental Studies, Ibaraki, Japan; 4 Department of Social and Preventive Epidemiology, School of Public Health, The University of Tokyo, Tokyo, Japan; 5 Institute for Advancement of Clinical and Translational Science, Kyoto University Hospital, Kyoto, Japan; 6 Division of Nutritional Physiology, Kagawa Nutrition University, Saitama, Japan; 7 Graduate School of Life Science, Kobe Women’s University, Kobe, Japan; 8 Department of Food Science and Nutrition, School of Food Science and Nutrition, Mukogawa Women’s University, Nishinomiya, Japan; 9 Department of Nutrition Science, University of Nagasaki, Nagasaki, Japan; 10 Department of Human Life and Science, Kagoshima Women’s College, Kagoshima, Japan; 11 Department of Nutritional Sciences, Nakamura Gakuen University, Fukuoka, Japan; 12 Department of Nutrition, Tenshi College, Hokkaido, Japan; 13 Research Support Center, Shizuoka General Hospital, Shizuoka, Japan; 14 Faculty of Nutrition, Kobe Gakuin University, Kobe, Japan

**Keywords:** Vitamin D deficiency, Sun exposure, Young Japanese women, Predictive scoring system

## Abstract

**Objective:**

Vitamin D deficiency (VDD) is common among young women and causes various health problems, including those that occur during pregnancy and childbirth. Thus, we investigated the risk factors for VDD in young Japanese women and developed a simple risk scoring system called Vitamin D Deficiency Predicting Scoring (ViDDPreS).

**Design:**

A cross-sectional study, using the following factors for multivariate logistic regression analysis to create the ViDDPreS score: residential area, season, cumulative ambient ultraviolet-B irradiation, BMI, vitamin D supplement use, sun exposure habits, frequency of habitual food intake and eating habits. The subjects were randomly divided into development and test sets for analysis. Serum 25-hydroxivitamin D concentration of less than 20 ng/ml was defined as VDD.

**Setting:**

Four regions (Hokkaido/Tohoku, Kanto, Chubu/Kinki/Shikoku and Kyushu/Okinawa) in Japan.

**Participants:**

Five hundred and eighty-three healthy women aged 18–40 years.

**Results:**

In the development set, the VDD group (68·4 %) had higher proportions of the following variables than the non-VDD group: residential area outside the Kanto region; blood samples obtained in winter; low BMI (<18·5 kg/m^2^); vitamin D supplement non-users; short time regularly spent outside on weekdays; intake of fish, vitamin D-abundant fish, dried fish and redfish less than once a week. VDD risk was classified as low, medium or high according to the ViDDPreS scores including these contributing factors, with a test set C-index of 0·671.

**Conclusion:**

We identified the risk factors for VDD in young Japanese women and developed a simple risk scoring system that enables us to assess VDD risk and aid in the development of appropriate prevention and treatment strategies for this population.

Vitamin D deficiency (VDD)/insufficiency is a risk factor for fractures, various non-communicable diseases and even infections^(1)^. Recently, maternal VDD has been associated with neonatal vitamin D status^(2)^, pre-eclampsia^(3)^, gestational diabetes^(4)^ and preterm birth or small birth for gestational age^(5)^. In addition, maternal vitamin D intervention increases circulating 25-hydroxyvitamin D (25(OH)D) levels in both pregnant women and infants, as well as the birth weight of newborns^(6)^. Nevertheless, a recent systematic review described a high global prevalence of VDD risk among pregnant women and newborns, with the percentage of pregnant women with serum 25-hydroxyvitamin D levels below 20 ng/ml being 64 % in the USA, 57 % in Europe, 46 % in the Eastern Mediterranean and 87 % in Southeast Asia, indicating that pregnant women in most regions have high prevalence of VDD^(7)^. In Japan, the majority of pregnant^(8)^ and non-pregnant young women reportedly have VDD^(9)^. Furthermore, regarding osteoporosis, a major non-communicable disease, peak bone mass in young adults is suggested to be associated with future risk of osteoporosis^(10)^. Since vitamin D plays a crucial role in bone formation^(10)^, preventing this disease is important regardless of childbearing status. Therefore, strategies to improve the vitamin D status of young women are important to prevent unfavourable health consequences for themselves and their offspring. To avoid VDD, the vitamin D nutritional status of the population should be determined first. The most common way to assess vitamin D nutritional status is to measure the circulating concentrations of 25(OH)D; however, this method incurs a cost of approximately 35 US dollars and involves venipuncture. The National Institute for Health and Care Excellence (NICE) reported the public health guideline, ‘Vitamin D: supplement use in specific population groups’, which states that prescribing inexpensive vitamin D supplements without assessing vitamin D levels would be more economical^(11)^. Since the usefulness of vitamin D intervention is limited to individuals with VDD^(12)^, it is desirable to develop a simple and low-cost method to assess vitamin D nutritional status.

Studies predicting vitamin D status have been conducted using self-administered questionnaires, and systematic reviews have been published^(13)^. However, studies that focus on VDD in young women are quite limited^(14)^.

These considerations led us to collect data on seasons and lifestyles, including sun exposure habits and dietary habits, associated with serum 25(OH)D concentrations in young Japanese women from four different latitudes, identify predictors of serum 25(OH)D concentrations and develop a predictive model for VDD.

## Methods

### Study design, places and timing

This study employed a cross-sectional design. Healthy young women aged 18–40 years residing in four regions in Japan were enrolled in the study from November 2020 to December 2022. The four regions include Hokkaido/Tohoku, Kanto, Chubu/Kinki/Shikoku and Kyushu/Okinawa, considering the influence of latitude on serum 25(OH)D concentrations. Detailed information on the prefectures and their latitudes included in this study was provided in our previous report^(15)^. This study was conducted during two seasons: summer (June–September in 2020 and 2022, and June–October in 2021) and winter (December–February), corresponding to the highest and lowest serum 25(OH)D values, respectively^(16)^.

### Participants and their data measurements

Students affiliated with nutrition departments were invited to participate in the study by a research coordinator from their respective universities, along with volunteers who agreed to participate.

The exclusion criteria were as follows: co-morbidities, such as severe chronic kidney disease, autoimmune disease, cancer, liver disease and diabetes mellitus. Those with restricted sun exposure due to diseases, such as xeroderma pigmentosum, and those with diseases possibly interfering with vitamin D absorption, such as chronic gastrointestinal disorders and cystic fibrosis, were also excluded.

The participants were asked to complete a questionnaire regarding their dietary history and habitual lifestyle during the week prior to blood collection. Blood collection was not restricted to fasting, was performed in the morning and was stored in a refrigerator at 4–10°C before centrifugation. Biochemical tests were performed the next day, and residual serum samples were stored at –80°C to measure the serum 25(OH)D concentration. Participants with missing data in the questionnaire were contacted, and the required information was obtained from all of them.

### Dietary assessment and variables

For dietary assessment, the Diet History Questionnaire (DHQ), a validated, comprehensive, self-administered, semi-quantitative questionnaire that assesses habitual nutrient intake, including vitamin D, was used over the month^(17–19)^. Ambiguity in responses was checked for and corrected by trained research staff.

Estimates of habitual daily intakes of energy, nutrients and food were calculated based on Japan’s Standard Tables of Food Composition^(20)^. The intake of nutrients, including vitamin D and foods, was adjusted using the density method and expressed as an amount (µg) per 4,184 kJ daily energy intake.^(21)^.

### Questionnaire on habitual lifestyles, such as sun exposure and sunscreen use

The questionnaire on habitual lifestyle is shown in Table 1. The investigation items included participant characteristics, habitual sun exposure and habitual dietary intake. These items were adapted from our previously developed ‘Vitamin D Deficiency Questionnaire for Japanese: VDDQ-J’^(22)^.

**Table 1:** Participant’s background in the development set

Variable	Category or (unit)	Serum total 25(OH)D < 20 ng/ml		Serum total 25(OH)D ≥ 20 ng/ml		
		*n* 199	sd or Q1-Q3, and %	*n* 92	sd or Q1-Q3, and %	*P*-value
Serum total 25(OH)D level (s d)	(ng/ml)	14·5	3·5	25·2	6·2	<0·001
Serum 25(OH)D_3_ level (s d)	(ng/ml)	12·7	3·2	22·1	5·3	<0·001
Serum 25(OH)D_2_ level (s d)	(ng/ml)	0·47	0·30	0·52	0·30	0·171
Serum 24,25(OH)_2_D_3_ level (s d)	(ng/ml)	1·32	0·47	2·55	1·17	<0·001
Serum iPTH level (s d)	(pg/ml)	36·8	13·3	32·8	10·4	0·015
The residential area	Kanto	29	14·6 %	34	37·0 %	<0·001
	Hokkaido/Tohoku	36	18·1 %	16	17·4 %	
	Chubu/Kinki/Shikoku	55	27·6 %	18	19·6 %	
	Kyushu/Okinawa	79	39·7 %	24	26·1 %	
Season of blood drawn	Winter (December–February)	108	54·3 %	39	42·4 %	0·060
	Summer (July–September)	91	45·7 %	53	57·6 %	
The UV-B irradiation 30 d (s d)	(100 mJ/cm^2^)	320·3	222·9	320·4	235·3	0·152
Age (s d)	(y)	20·8	2·3	20·7	2·1	0·651
BMI (s d)	(kg/m^2^)	20·5	2·3	21·3	2·9	0·020
Alcohol consumption	More than 4 times per week	3	2·2 %	2	2·9 %	0·662
	Less than once per month	60	43·2 %	25	36·8 %	
	Never	76	54·7 %	41	60·3 %	
Use of supplement containing vitamin D	No	194	97·5 %	81	88·0 %	0·001
	Yes	5	2·5 %	11	12·0 %	
Exercise habit	More than twice per week	11	5·5 %	7	7·6 %	0·698
	Once per week	31	15·6 %	14	15·2 %	
	1–2 times per month	43	21·6 %	24	26·1 %	
	Rarely	114	57·3 %	47	51·1 %	
Suntan within the past 12 months	No	101	50·8 %	42	45·7 %	0·418
	Yes	98	49·2 %	50	54·3 %	
Sun exposure in the last 3 months	Always	17	8·5 %	5	5·4 %	0·589
	More often than not	41	20·6 %	24	26·1 %	
	Sometimes	29	14·6 %	17	18·5 %	
	Infrequently	42	21·1 %	16	17·4 %	
	Never	70	35·2 %	30	32·6 %	
Time regularly spent outside on weekday	(min/d)	35	15–60	47·5	30–70	0·041
Time spent outside on weekdays	≥3 h/d	8	4·0 %	3	3·3 %	0·247
	2–<3 h/d	9	4·5 %	6	6·5 %	
	1–<2 h/d	33	16·6 %	24	26·1 %	
	<1 h/d	126	63·3 %	53	57·6 %	
	Rarely	23	11·6 %	6	6·5 %	
Time regularly spent outside on weekends	(min/d)	25	10–45	30	10–55	0·210
Time spent outside on weekends	≥3 h/d	9	4·5 %	5	5·4 %	
	2–<3 h/d	27	13·6 %	22	23·9 %	0·201
	1–<2 h/d	15	7·5 %	5	5·4 %	
	<1 h/d	103	51·8 %	38	41·3 %	
	rarely	45	22·6 %	22	23·9 %	
Sunscreen use	Always	55	27·6 %	22	23·9 %	0·762
	More often than not	34	17·1 %	21	22·8 %	
	Sometimes	31	15·6 %	14	15·2 %	
	Infrequently	29	14·6 %	15	16·3 %	
	Never	50	25·1 %	20	21·7 %	
Sunscreen use for arms and legs	No	83	41·7 %	39	42·4 %	0·913
	Yes	116	58·3 %	53	57·6 %	
Paying attention to UV exposure	No	76	38·2 %	38	41·3 %	0·613
	Yes	123	61·8 %	54	58·7 %	
Recently wearing clothes	Arms and legs exposed	123	61·8 %	50	54·3 %	0·228
	Without skin exposed	76	38·2 %	42	45·7 %	
Skin type	V and VI: Dark brown or black skin	45	22·6 %	25	27·2 %	0·065
	IV: Light brown skin (burns minimally, tans easily)	14	7·0 %	4	4·3 %	
	III: Darker white skin (tans after initial burn)	71	35·7 %	25	27·2 %	
	II: Fair skin, blue eyes (burns easily, tans poorly)	51	25·6 %	35	38·0 %	
	I: Pale white skin, blue/green eyes, blond/red hair (always burns, does not tan)	18	9·0 %	3	3·3 %	
Habitual intake of fish	Less than 1 d a week	58	29·1 %	16	17·4 %	0·004
	1–2 d a week	76	38·2 %	28	30·4 %	
	2–3 d a week	63	31·7 %	43	46·7 %	
	More than 4 d a week	2	1·0 %	5	5·4 %	
Habitual intake of the vitamin D-abundant fish (e.g. salmon, sardine, saury, flounder, eel, herring and grunt)	Less than 1 d a week	126	63·3 %	41	44·6 %	0·005
	1–2 d a week	57	28·6 %	32	34·8 %	
	2–3 d a week	15	7·5 %	18	19·6 %	
	More than 4 d a week	1	0·5 %	1	1·1 %	
Frequency of low-fat milk intake	More than once a week	22	11·1 %	13	14·1 %	0·463
	Less than once a week	176	88·9 %	79	85·9 %	
Frequency of milk intake	More than once a week	79	39·7 %	40	43·5 %	0·542
	Less than once a week	120	60·3 %	52	56·5 %	
Frequency of yogurt intake	More than once a week	103	52·0 %	57	62·6 %	0·092
	Less than once a week	95	48·0 %	34	37·4 %	
Frequency of cheese intake	More than once a week	71	35·7 %	41	44·6 %	0·147
	Less than once a week	128	64·3 %	51	55·4 %	
Frequency of cottage cheese intake	More than once a week	3	1·5 %	2	2·2 %	0·684
	Less than once a week	196	98·5 %	90	97·8 %	
Frequency of milk beverage drink	More than once a week	23	11·6 %	11	12·0 %	0·922
	Less than once a week	176	88·4 %	81	88·0 %	
Frequency of butter intake	More than once a week	41	20·6 %	25	27·2 %	0·213
	Less than once a week	158	79·4 %	67	72·8 %	
Frequency of margarine intake	More than once a week	29	14·6 %	14	15·2 %	0·886
	Less than once a week	170	85·4 %	78	84·8 %	
Frequency of dried fish intake	More than once a week	12	6·0 %	11	12·0 %	0·082
	Less than once a week	187	94·0 %	81	88·0 %	
Frequency of fish eaten with the bone intake	More than once a week	17	8·5 %	8	8·7 %	0·966
	Less than once a week	182	91·5 %	84	91·3 %	
Frequency of tuna intake	More than once a week	22	11·1 %	11	12·0 %	0·822
	Less than once a week	177	88·9 %	81	88·0 %	
Frequency of eel intake	More than once a week	0	0·0 %	1	1·1 %	0·141
	Less than once a week	199	100·0 %	91	98·9 %	
Frequency of white fish intake	More than once a week	31	15·6 %	24	26·1 %	0·033
	Less than once a week	168	84·4 %	68	73·9 %	
Frequency of blueback fish intake	More than once a week	46	23·1 %	27	29·3 %	0·254
	Less than once a week	153	76·9 %	65	70·7 %	
Frequency of redfish intake	More than once a week	48	24·1 %	40	43·5 %	0·001
	Less than once a week	151	75·9 %	52	56·5 %	
Frequency of egg	More than once a week	186	93·5 %	84	91·3 %	0·507
	Less than once a week	13	6·5 %	8	8·7 %	
Frequency of mushrooms	More than once a week	141	70·9 %	64	69·6 %	0·823
	Less than once a week	58	29·1 %	28	30·4 %	

25(OH)D, 25-hydroxyvitamin D; 24,25(OH)_2_D_3_, 24,25-dihydroxycholecalciferol; iPTH, intact parathyroid hormone; UV-B, ultraviolet B.

The frequency of intake of each food item was expressed as eight categories (at least twice a day, once a day, 4–6 times a week, 2–3 times a week, once a week, 2–3 times a month, once a month and not at all) and was reclassified into two categories: more than once a week and less than once a week.

Continuous variables are expressed as mean (sd) or median (Q1–Q3), and categorical variables are expressed as frequency (percentage) using the Wilcoxon rank-sum test for continuous variables and the *χ* ^2^ test for categorical variables.

### Serum 25(OH)D concentration-related variables as outcomes

Serum vitamin D metabolites, 25(OH)D_2_, 25(OH)D_3_ and 24,25-dihydroxycholecalciferol (24,25(OH)_2_D_3_), were measured using modified liquid chromatography-tandem mass spectrometry (LC-APCI-MS/MS) method^(23,24)^. Their sum was used to calculate total serum 25(OH)D levels.

All the serum samples were collected from a single laboratory. SRM 972a (National Institute of Standards and Technology, NIST), which consists of four vials of level 1–4 frozen serum from healthy donors with different 25(OH)D concentrations, was used to estimate the accuracy. The results satisfied the criteria for the LC-MS/MS performance threshold of the AOAC procedure for 25(OH)D standardisation/validation (VDSP)^(25)^. Details are provided in the Supplementary Text.

The consensus is that serum 25(OH)D concentrations <20 ng/ml, 20–30 ng/ml and ≥30 ng/ml indicate VDD, vitamin D insufficiency and vitamin D sufficiency, respectively^(26)^. On the basis of this consensus, this study defined VDD as a serum 25(OH)D concentration of less than 20 ng/ml.

Additionally, serum intact parathyroid hormone, a surrogate marker of 25(OH)D, was measured using an electrochemiluminescence immunoassay (SRL, Inc.).

### Estimation of the cumulative ambient ultraviolet-B irradiation

We calculated the ultraviolet-B (UV-B) radiation flux density using a radiative-transfer code called SMARTS2 (Simple Model of the Atmospheric Radiative Transfer of Sunshine, version 2), developed by Gueymard, to calculate the flux density of solar radiation on the ground surface E(λ) for wavelengths between 280 and 4000 nm^(27)^. Based on this, we estimated the cumulative ambient UV-B irradiation from 1 month to 1 d before blood was drawn. Details are provided in the Supplementary Text.

### Predictive factor candidates

The candidate predictive variables were as follows: age, residential area, skin type, smoking status, medication history, habitual drinking, physical activity, season of blood collection, habitual sun exposure time and frequency, sunscreen use, experience of suntan within the past 12 months, vitamin D supplement use, habitual avoidance of sun exposure with parasol use or wearing long-sleeved clothes, weekly frequency of vitamin D-abundant fish consumption, DHQ’s weekly frequency of milk and dairy products, fish (dried fish, fish eaten with the bones, tuna, eel, white fish, blueback fish and redfish), egg, and mushroom, and cumulative ambient UV-B irradiation.

### Statistical analysis

Prior to commencing the research, the necessary sample size was determined to be 600 cases based on a prior study indicating that 52 % of females between 17 and 18 years of age had VDD^(28)^. Assuming that half of the study participants also had VDD, we anticipated 300 cases of VDD. We also projected that 30 % of the participants would be lost owing to incomplete data or withdrawal of consent.

Continuous variables were expressed as mean (sd) or median (Q1–Q3), and categorical variables were expressed as frequencies (percentages). The Wilcoxon rank-sum test for continuous variables and the *χ* ^2^ test for categorical variables were used to compare the two groups.

Half of the data was designated as the development set for model construction, with the remaining portion serving as a validation set to assess the model’s performance. In the development set, variables with a *P*-value <0·1 in the univariable analysis as well as previously reported predictors were included in the multivariable logistic regression model to construct the predictive score. OR and 95 % CI were estimated using a logistic regression model. For data with an absolute Spearman’s correlation coefficient > 0·4, one of the two variables was not used in the multivariable model because of possible multicollinearity. The regression coefficients were multiplied by a constant number and converted into integral numbers as predictive scores. In the multivariate model, no variable selection based on the *P*-value was performed^(29)^. The predictive performance was checked using the C-index in the test set. This process is reported in accordance with the TRIPOD statement^(30)^.

Missing data were not imputed. All analyses were considered statistically significant at *P* < 0·05 (two-tailed). All the analyses were performed using SAS version 9.4 (SAS Institute).

### Ethics and study registration

Participants who voluntarily provided written consent to participate in this study were enrolled. This study conformed to the Ethical Principles for Medical Research Involving Human Subjects issued by the Ministry of Health, Labour, and Welfare and Ministry of Education, Culture, Sports, Science, and Technology in Japan. The study protocol was approved by the ethics committee of each institution. The details were provided in our previous report^(15)^. This study was registered with the UMIN-CTR (ID: UMIN000041527).

## Results

### Study participants

A total of 589 healthy women aged 18–40 living in Japan were enrolled in this study. Ultimately, 583 participants with measured serum 25(OH)D levels were included in the analysis. There were no differences between the participants’ characteristics in the development (*n* 291) and test sets (*n* 292), which were randomly partitioned, except for exercise habits and time spent outside on weekdays (see online supplementary material, Supplementary Table 1).

### Characteristics of participants

In the development set, no significant differences were observed in the amount of UV-B irradiation 30 d prior to blood collection, although there was a higher percentage of winter blood draws in the VDD group than in the non-VDD group, without a significant difference (*P* = 0·060). A total of sixteen (5·5 %) participants consumed vitamin D supplements. In both groups, more than half of the participants had little or no exercise habits, and sun exposure was infrequent. The BMI was in the low to normal weight range in both groups. The frequency of habitual intake of vitamin D-rich fish was low in both groups (Table 1). The VDD group had higher proportions of the following variables than the non-VDD group: residential area outside the Kanto region; non-vitamin D supplement users; short time regularly spent outside on weekdays; and less than once a week intake of fish, vitamin D-abundant fish, white fish and redfish. The BMI in the VDD group was lower than that in the non-VDD group (Table 1). Serum 25(OH)D_2_ concentrations did not differ between the two groups; however, 25(OH)D_3_ and 24,25(OH)_2_D_3_ levels were higher in the non-VDD group. In contrast, the serum intact parathyroid hormone (iPTH) levels were higher in the non-VDD group.

In terms of nutrient intake, the VDD group had lower intakes of protein, Ca and vitamin D than the non-VDD group, both in crude values and per 4,184 kJ. The VDD group had a lower fish intake than the non-VDD group for both crude and energy-adjusted values, lower milk intake for crude value and lower egg intake for energy-adjusted values (see online supplementary material, Supplementary Table 2). The percentage of participants with vitamin D intake above the Adequate Intake in Dietary Reference Intakes in Japan was lower in the VDD group (7·5 %) than in the non-VDD group (22·8 %).

### Predictive factors of vitamin D deficiency

The results of the univariate model for the development set are shown in Table 2. The results of the multivariable analysis showed that the following factors are predictive of VDD: residential area outside the Kanto region (Kyushu/Okinawa: OR: 3·86 (95 % CI 1·87, 7·94), Chubu/Kinki/Shikoku: 3·10 (1·43, 6·73), Hokkaido/Tohoku: 2·80 (1·22, 6·42)), blood samples obtained in winter (1·65 (0·95, 2·86)), lower BMI (<18·5 kg/m^2^) (3·06 (1·16, 8·06)), non-vitamin D supplement users (9·15 (2·84, 29·45)), time regularly spent outside of less than 60 min on weekdays (1·63 (0·89, 2·97)) and less than once a week intake of vitamin D-abundant fish (2·26 (1·29, 3·96)), dried fish (1·26 (0·50, 3·20)) (Table 3).

**Table 2:** Univariable regression model for VDD in the development set

Variable	Category or (unit)	Univariable regression model			
		OR	95 % CI	*P*-value	*P*-value (global)
The residential area	Kanto	1			<0·001
	Hokkaido/Tohoku	2·64	1·22, 5·70	0·014	
	Chubu/Kinki/Shikoku	3·58	1·73, 7·41	0·001	
	Kyushu/Okinawa	3·86	1·97, 7·57	<0·001	
Season of blood drawn	Winter (December–February)	1			0·060
	Summer (July–September)	1·61	0·98, 2·66	0·060	
Age	(y)	1·03	0·92, 1·16	0·618	0·618
BMI	(kg/m^2^)	0·88	0·80, 0·97	0·013	0·013
BMI category	≥18·5 kg/m^2^	1			0·029
	<18·5 kg/m^2^	2·59	1·11, 6·08	0·029	
Alcohol consumption	More than 4 times per week	1			0·662
	Less than once per month	1·6	0·25, 10·17	0·618	
	Never	1·24	0·20, 7·70	0·821	
Use of supplements containing vitamin D	No	1			0·003
	Yes	0·19	0·06, 0·56	0·003	
Exercise habit	More than twice per week	1			0·700
	Once per week	1·41	0·45, 4·40	0·555	
	1–2 times per month	1·14	0·39, 3·33	0·810	
	Rarely	1·54	0·56, 4·22	0·398	
Suntan within the past 12 months	No	1			0·419
	Yes	1·23	0·75, 2·01	0·419	
Sun exposure in the last 3 months	Always	1			0·594
	More often than not	0·5	0·16, 1·54	0·228	
	Sometimes	0·5	0·16, 1·61	0·245	
	Infrequently	0·77	0·24, 2·44	0·660	
	Never	0·69	0·23, 2·03	0·497	
Time regularly spent outside on weekdays	(min)	1·00	0·99, 1·00	0·531	0·531
Time regularly spent outside on weekdays category	≥60 min	1			0·156
	<60 min	1·46	0·86, 2·48	0·156	
Time spent outside on weekdays	<1 h	1			0·060
	≥1 h	0·60	0·35, 1·02	0·060	
Time regularly spent outside on weekends	(min)	1·00	0·99, 1·00	0·539	0·539
Time regularly spent outside on weekends category	≥60 min	1			0·346
	<60 min	1·33	0·73, 2·42	0·346	
Time spent outside on weekends	<1h	1			0·109
	≥1 h	0·65	0·38, 1·10	0·109	
Sunscreen use	Always	1			0·764
	More often than not	0·65	0·31, 1·35	0·247	
	Sometimes	0·89	0·40, 1·98	0·767	
	Infrequently	0·77	0·35, 1·71	0·527	
	Never	1	0·49, 2·05	>0·999	
Sunscreen use for arms and legs	No	1			0·913
	Yes	0·97	0·59, 1·60	0·913	
Paying attention to UV exposure	No	1			0·613
	Yes	0·88	0·53, 1·45	0·613	
Recently wearing clothes	Arms and legs exposed	1			0·229
	Without skin exposed	0·74	0·45, 1·21	0·229	
Skin type	VI: Dark brown or black skin	1			0·075
	IV: Light brown skin (burns minimally, tans easily)	1·94	0·58, 6·55	0·283	
	III: Darker white skin (tans after initial burn)	1·58	0·81, 3·08	0·181	
	II: Fair skin, blue eyes (burns easily, tans poorly)	0·81	0·42, 1·55	0·525	
	I: Pale white skin, blue/green eyes, blond/red hair (always burns, does not tan)	3·33	0·89, 12·43	0·073	
Habitual intake of fish	<once a week	1			0·034
	≥once a week	0·51	0·28, 0·95	0·034	
Habitual intake of the vitamin D abundant fish	<once a week	1			0·003
	≥once a week	0·47	0·28, 0·77	0·003	
Frequency of low-fat milk intake	<once a week	1			0·464
	≥once a week	1·32	0·63, 2·75	0·464	
Frequency of milk intake	<once a week	1			0·542
	≥once a week	1·17	0·71, 1·93	0·542	
Frequency of yogurt intake	<once a week	1			0·093
	≥once a week	1·55	0·93, 2·57	0·093	
Frequency of cheese intake	<once a week	1			0·148
	≥once a week	1·45	0·88, 2·40	0·148	
Frequency of cottage cheese intake	<once a week	1			0·686
	≥once a week	1·45	0·24, 8·84	0·686	
Frequency of milk beverage drink	<once a week	1			0·921
	≥once a week	1·04	0·48, 2·23	0·921	
Frequency of butter intake	<once a week	1			0·215
	≥once a week	1·44	0·81, 2·55	0·215	
Frequency of margarine intake	<once a week	1			0·885
	≥once a week	1·05	0·53, 2·10	0·885	
Frequency of dried fish intake	<once a week	1			0·087
	≥once a week	2·12	0·90, 5·00	0·087	
Frequency of fish eaten with the bone’s intake	<once a week	1			0·965
	≥once a week	1·02	0·42, 2·46	0·965	
Frequency of tuna intake	<once a week	1			0·822
	≥once a week	1·09	0·51, 2·36	0·822	
Frequency of eel intake	<once a week	1			NE
	≥once a week	NE	NE	NE	
Frequency of white fish intake	<once a week	1			0·035
	≥once a week	1·91	1·05, 3·50	0·035	
Frequency of blueback fish intake	<once a week	1			0·255
	≥once a week	1·38	0·79, 2·41	0·255	
Frequency of redfish intake	<once a week	1			0·001
	≥once a week	2·42	1·43, 4·09	0·001	
Frequency of egg	<once a week	1			0·509
	≥once a week	0·73	0·29, 1·84	0·509	
Frequency of mushrooms	<once a week	1			0·823
	≥once a week	0·94	0·55, 1·61	0·823	
The UV-B irradiation (100 mJ/cm^2^) 30 d		0·93	0·83, 1·03	0·152	0·152
The UV-B irradiation (100 mJ/cm^2^) 21 d		0·93	0·84, 1·03	0·145	0·145
The UV-B irradiation (100 mJ/cm^2^) 14 d		0·93	0·85, 1·02	0·108	0·108
The UV-B irradiation (100 mJ/cm^2^) 7 d		0·93	0·85, 1·01	0·074	0·074
The UV-B irradiation (100 mJ/cm^2^) 3 d		0·94	0·87, 1·02	0·139	0·139

NE, not valuable; VDD, vitamin D deficiency; UV-B, ultraviolet B.

Participants were asked about the time spent outside (min) on weekdays and weekends between 7:00 a.m. and 6:00 p.m. in 10-min increments. Data were dichotomised into less-than-60 min and more-than-60-min groups.

**Table 3:** Multivariable regression model for VDD in the development set

Variable (reference)	Category	Multivariable regression model (*n* 291)			C-index
		OR	95 % CI	*P*-value	
**Multivariable regression model**					
The residential area (Kanto region)	Kyushu/Okinawa	3·86	1·87, 7·94	<0·001	0·757
	Chubu/Kinki/Shikoku	3·10	1·43, 6·73	0·004	
	Hokkaido/Tohoku	2·80	1·22, 6·42	0·015	
Season of blood drawn (Summer (July–September))	Winter (December–February)	1·65	0·95, 2·86	0·075	
BMI category (≥18·5 kg/m^2^)	<18·5 kg/m^2^	3·06	1·16, 8·06	0·023	
Use of supplements containing vitamin D (Yes)	No	9·15	2·84, 29·45	<0·001	
Time regularly spent outside on weekdays (≥60 min)	<60 min	1·63	0·89, 2·97	0·112	
Frequency of vitamin D abundant fish intake (≥once a week)	<once a week	2·26	1·29, 3·96	0·005	
Frequency of dried fish intake (≥once a week)	<once a week	1·26	0·50, 3·20	0·630	
**Risk scoring tool**					
Risk score	1 point	1·31	1·20, 1·42	<0·001	0·748
Risk classification	0–13 points	1			0·730
	14–17 points	2·81	1·46, 5·40	0·002	
	18–24 points	9·50	4·79, 18·84	<0·001	

VDD, vitamin D deficiency.

Owing to multicollinearity, skin type, habitual fish intake, white fish, red fish and 7-d cumulative ambient UV-B irradiation were excluded from the multivariate regression model. The frequency of yogurt consumption was excluded from the model because its score was 0.

### Scoring and classification for vitamin D deficiency risk predicting

**Fig. 1:**
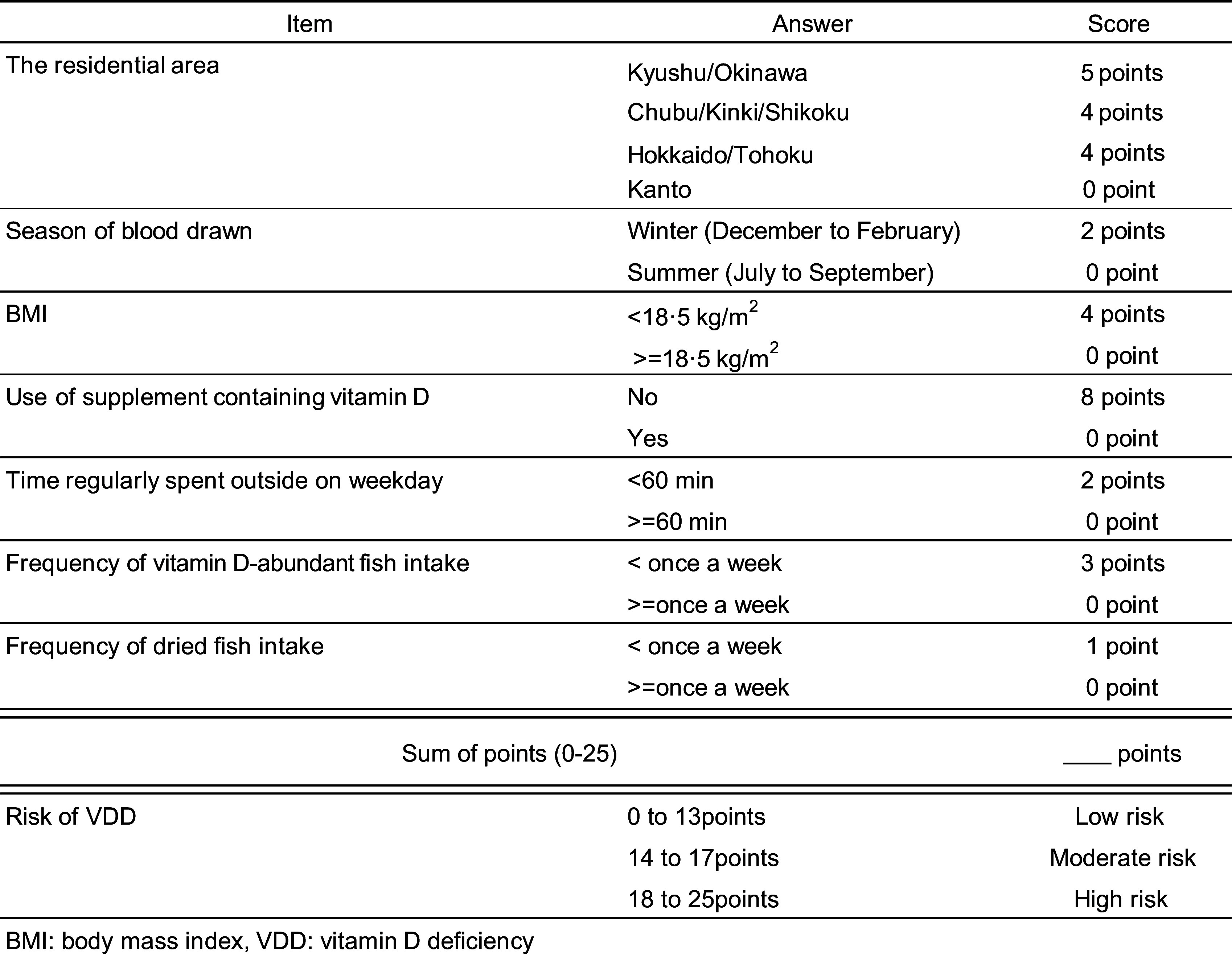
Risk score calculation form. The regression coefficients, which were determined as predictors of VDD risk, were multiplied by a constant number and converted into integers as the predictive scores

Based on the multivariate regression model in the development set (Table 3), a scoring table was created to estimate the VDD risk scores (Fig. 1). The OR (95 % CI) for one increment in the risk score was 1·31 (1·20, 1·42). The 0–13, 14–17 and 18–24 points represent low, moderate and high risks of VDD, respectively. The OR (95 % CI) for the moderate and high risk classification compared with low were, respectively, 3·19 (1·56, 6·52) and 6·44 (3·18, 13·06).

**Fig. 2:**
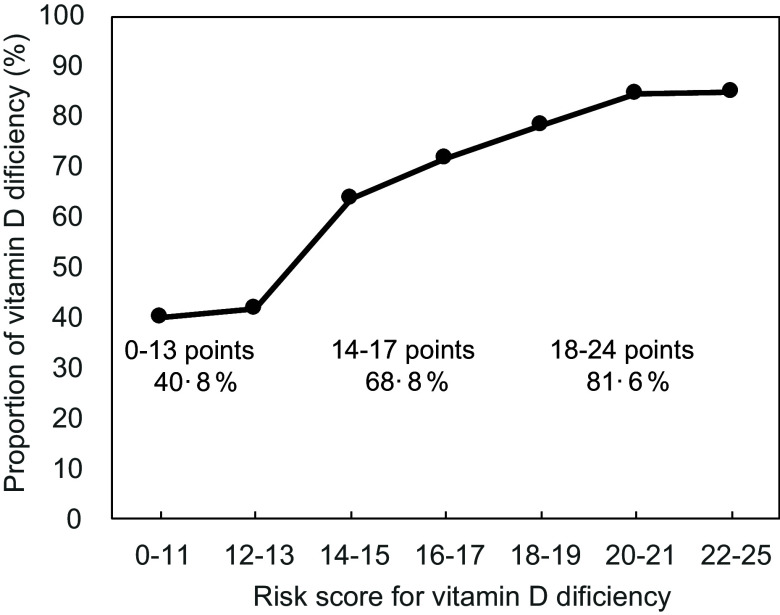
Proportion of vitamin D deficiency in each risk score group in the test set. The proportion of VDD was shown in each score category

In the test set, the C-index, which indicates the predictive performance, of the risk scores and risk classification were 0·690 and 0·671, respectively (Table 4). The proportion of VDD in the score category is shown in Fig. 2, where the percentage of VDD increases as the class increases. The percentage of participants with VDD was 68·4 % in the development set and 70·5 % in the test set, with no significant difference between the two groups (see online supplementary material, Supplementary Table 1).

**Table 4:** Evaluation of risk scoring tool for VDD in the test set

Variable	Category or unit			Multivariable regression model (*n* 292)			C-index
		Frequency of VDD		OR	95 % CI	*P*-value	
Risk scoring tool							
Risk score	1 point			1·22	1·13, 1·32	<0·001	0·690
Risk classification	0–13 points	20/49	40·8%	1			0·671
	14–17 points	66/96	68·8%	3·19	1·56, 6·52	0·002	
	18–24 points	120/147	81·6%	6·44	3·18, 13·06	<0·001	

VDD, vitamin D deficiency.

## Discussion

In this cross-sectional study, the multivariable model identified seven variables associated with the prevalence of VDD: residing outside the Kanto region, blood samples obtained during winter, low BMI, not using vitamin D supplements, spending less than 60 min outside on weekdays, consuming vitamin D-rich fish less than once a week and consuming dried fish less than once a week. We created a score sum from these seven variables to predict VDD and built a scoring system with high predictive performance, the Vitamin D Deficiency Predicting Scoring (ViDDPreS).

During the initial preparation of the protocol, we expected that 30 % of the subjects would be lost due to incomplete datasets or withdrawal of consent and planned to enrol 600 participants with 50 % of the VDD subjects^(15)^. A total of 583 subjects, of whom 405 had VDD, were included in the analysis, fulfilling the required sample size.

In the present study, 69·5 % of the subjects had VDD. In a survey of 296 young Japanese women, approximately 64 % had VDD^(9)^. Additionally, in our previous study of female university students with a low frequency of sun exposure, 81·5 % of the participants were found to have VDD^(31)^. Thus, the vitamin D status of the current subjects likely did not deviate from that of healthy young Japanese women, and our results can be generalised.

Among the four regions, the serum 25(OH)D concentration was the highest in the Kanto region and lowest in the Kyushu region, although the average UV irradiation 30 d before blood collection was the highest in the latter. In contrast, the vitamin D intake was higher in the Kanto region than in any other area (*P* = 0·011, data not shown). Therefore, individual factors, such as sun exposure habits and vitamin D intake status, were considered to affect vitamin D status more strongly than regional, latitude-dependent factors, such as the difference in UV exposure.

In previous studies, the season of blood collection was a predictor of VDD prevalence^(22,32,33)^. Although the season is an important determinant of UV exposure, data from only two seasons (summer and winter) were available in the present study. The prediction models using either the season of blood collection or UV-B irradiation doses yielded similar predictive performances (Table 3 and model 3 of online supplementary material, Supplementary Table 3). We chose the season rather than UV-B exposure as the main multivariate model (Table 3).

Most previous studies have shown that a higher BMI corresponding to obesity is a risk factor for VDD^(33–38)^. In the current study, however, BMI less than 18·5 kg/m^2^ was a risk factor for VDD. This discrepancy may arise from the fact that obesity is far less frequent in young Japanese women than in American or European women. Nevertheless, in a study of 348 young Australian women (mean BMI 23·5 ± 4·1 kg/m^2^), serum 25(OH)D levels were lower in the underweight group^(14)^. However, this requires further investigation.

The study showed that participants taking vitamin D supplements were less likely to have VDD prevalence, which is consistent with the previous reports^(34,37–39)^. However, other studies have reported that vitamin D supplementation did not contribute to the prevalence of VDD^(40,41)^. This discrepancy is attributable to two factors. First, in studies reporting no effects of supplementation, the percentage of supplement users was low at only 6 %^(40)^ and 3·4 %^(41)^. Second, vitamin D-fortified foods are widely used in many countries, the intake of which will markedly improve vitamin D status and may obscure the effects of vitamin D supplementation. In Japan, where food fortification with vitamin D is uncommon, the effects of vitamin D supplementation would be more marked, which is likely to be the basis for the positive effects of vitamin D supplementation, despite the low percentage of supplement users in the current study.

The present study found that spending less than 60 min outside on weekdays was related to the prevalence of VDD, which is consistent with many previous reports that sun exposure habit has been found to be a predictor of VDD^(22,34,35,37–39,41,42)^. In contrast, this study found no relationship between sunscreen use and vitamin D levels. Although this result may be paradoxical, previous studies have reported that sunscreen use does not affect serum 25(OH)D levels^(43,44)^, whereas others have suggested that sunscreen may inhibit vitamin D production^(45)^. In addition, no association was observed between sunscreen use and serum 25(OH)D concentration in a cross-sectional study, and higher levels of 25(OH)D were reported in sunscreen users^(46)^. These paradoxical results presumably reflect the fact that the effect of UV irradiation from sun exposure outweighs the UV protective effect of inadequately applied sunscreens.

Our study indicated that fish intake (vitamin D-abundant and dried fish) less than once a week contributes to VDD risk. The frequency of fish intake has been reported to contribute to serum 25(OH)D concentration in Japanese subjects^(47)^. Regarding the relationship between vitamin D intake and serum 25(OH)D levels, vitamin D intake was positively associated with serum 25(OH)D concentration (*r* = 0·206, 95 % CI 0·093, 0·313, data not shown) in our study. Interestingly, pregnant Japanese women showed a positive relationship between energy-adjusted vitamin D intake and serum 25(OH)D concentrations (*r* = 0·304, *P* = 0·001) in winter, when serum 25(OH)D concentrations were less likely to be affected by sunlight^(19)^. Since fish and shellfish accounted for 79 % of the total vitamin D intake in the 2019 National Health and Nutrition Survey of Japan, it is conceivable that lower fish intake is a contributing factor to VDD. In other countries, milk, breakfast cereal and margarine are the main sources of vitamin D because they are fortified with vitamin D. However, these items are not fortified in Japan, and eggs are the second largest source of vitamin D after fish.

In summary, although previously reported factors contributing to the risk of VDD were significant contributing variables, our study is unique in showing a relatively large contribution of vitamin D intake from fish, as the intake status of fortified foods and supplements differs from that in other countries. Most previous screening tools have been used in older adults, and there are a limited number of screening methods for adults^(14,36,42)^. Furthermore, the known risk factors for VDD were identified. Although it is widely recognised that each of these factors is important, our approach using linear model prediction shows that these known risk factors contribute independently, and in combination, provide an assessment of VDD risk. As a known risk factor, VDD has been developed as an interpretable risk assessment tool, providing an intuitive tool for predicting and managing VDD risk from a public health perspective.

The C-index in the test set of the developed scoring tool for VDD was 0·690 for risk score and 0·671 for risk score classification. Since a C-index of 0·5 is not discriminable, 0·7–0·8 is acceptable, 0·8–0·9 is excellent, and 0·9 and above is generally considered excellent^(48)^, the developed risk scoring tool was determined to have adequate validity.

As noted earlier in the ‘Introduction,’ assessing vitamin D nutritional status by measuring circulating 25(OH)D concentrations is expensive and inappropriate for a population-level approach. The tool we developed can estimate the VDD risk by answering a simple question. Thus, the target population for a high-risk approach can be selected at low cost. In addition, if the tool is used in a population of young women, data on the actual VDD risk in young women can be easily collected, which can help in the search for effective interventions for young women (e.g. increased sun exposure and mechanisms to increase intake) and accelerate the development of nutrition policies. It would also help explore the appropriate use of currently underutilised vitamin D-fortified foods and other products when vitamin D intake is inadequate in a regular diet. On the other hand, the NICE reported that although subjects with severe VDD should have their circulating 25(OH)D levels measured, the cost-effectiveness of improving VDD was only examined under the condition that none of the participants had their circulating 25(OH)D levels measured^(11)^. Our screening tool can also be used to select participants for whom blood 25(OH)D levels should be measured. Furthermore, it can be used at the individual level to help identify the appropriate use of supplements and avoid unnecessary usage.

The present study has some limitations. First, because the survey was conducted among nutrition students with a high level of health literacy, the results may not be directly applicable to young women. However, this property of the participants can be advantageous in that highly accurate data can be obtained from nutrition students with high health literacy. Second, the participants lived under lifestyle restrictions to prevent the spread of COVID-19. Participants in the present study were asked to recall the same questionnaire form used in this study regarding their lifestyle before the COVID-19 pandemic. There was no significant difference in the sun exposure status before and after the pandemic (data not shown). Therefore, the target population had limited opportunities for sun exposure even before the COVID-19 pandemic, and lifestyle changes were unlikely. Third, our study included only summer and winter surveys. Therefore, a sensitivity analysis was conducted on the development set without the seasons of blood collection, and the C-index was 0·750 (see online supplementary material, Supplementary Table 3), which was similar to the results in Table 4. Therefore, even if data were available only for the summer and winter seasons, the impact would be negligible.

Fourth, SNP associated with serum 25(OH)D levels were not investigated. Although SNP in four genes related to vitamin D production and metabolism were identified in a previous study, they explained only 2·4 % of the variance in serum 25(OH)D levels^(49)^. Additionally, a recent study identified more than 150 related SNP that could explain 10·5 % of the variance in serum 25(OH)D levels^(12)^. Thus, the contribution of currently available SNP to serum 25(OH)D concentrations is not very high and is likely to be far less than that of sunlight exposure or vitamin D intake.

## Conclusions

VDD in young women is related not only to environmental factors such as latitude and region of residence but also to lower BMI, non-vitamin D supplement users, less sun exposure habits and lower frequency of fish consumption, and the VDD risk screening form ‘ViDDPreS’ based on these factors has a certain predictive performance and is expected to be useful for disease prevention through improvement of VDD.

## Supporting information

Kuwabara et al. supplementary material 1 Kuwabara et al. supplementary material

Kuwabara et al. supplementary material 2 Kuwabara et al. supplementary material

## Supplementary material

For supplementary material accompanying this paper visit https://doi.org/10.1017/S1368980024001708
