# Supplementary material for: Development of a predictive scoring system for vitamin D deficiency ‘Vitamin D Deficiency Predicting Scoring (ViDDPreS)’ based on the vitamin D status in young Japanese women: a nationwide cross-sectional study
Source: Public Health Nutr. 2024 Sep 27;27(1):e185. doi: 10.1017/S1368980024001708 (PMC11505081; doi:10.1017/S1368980024001708)
Supplement: Kuwabara et al. supplementary material 1 — Kuwabara et al. supplementary material [file S1368980024001708sup001.docx]

**Supplementary Table 1.** Comparison of participants distribution between development and test sets

| **Variable** | **Category or (unit)** | **Development set** | **Test set** | **p-value** |
| --- | --- | --- | --- | --- |
|  |  | **n=291** | **n=292** |  |
| Serum total 25(OH)D level | 25(OH)D <20 | 199 (68.4%) | 206 (70.5%) | 0.571 |
|  | 25(OH)D >=20 | 92 (31.6%) | 86 (29.5%) |  |
| Serum 25(OH)D_3_ level | (ng/mL) | 15.7 (SD: 6.0) | 15.4 (SD: 5.5) | 0.602 |
| Serum 25(OH)D_2_ level | (ng/mL) | 0.49 (SD: 0.30) | 0.49 (SD: 0.65) | 0.313 |
| Serum 24,25(OH)_2_D_3_ level | (ng/mL) | 1.71 (SD: 0.96) | 1.64 (SD: 0.76) | 0.495 |
| Serum iPTH level | (pg/mL) | 35.6 (SD: 12.5) | 37.0 (SD: 14.0) | 0.190 |
| The residential area | Kanto | 63 (21.6%) | 50 (17.1%) | 0.433 |
|  | Hokkaido/Tohoku | 52 (17.9%) | 49 (16.8%) |  |
|  | Chubu/Kinki/Shikoku | 73 (25.1%) | 74 (25.3%) |  |
|  | Kyushu/Okinawa | 103 (35.4%) | 119 (40.8%) |  |
| Season of blood drawn | Winter  (December to February) | 144 (49.5%) | 151 (51.7%) | 0.591 |
|  | Summer  (July to September) | 147 (50.5%) | 141 (48.3%) |  |
| The UV-B irradiation 30-day | (100 mJ/cm^2^) | 333.6 (SD: 231.9) | 337.0 (SD: 220.8) | 0.742 |
| Age | (y) | 20.8 (SD: 2.2) | 21.1 (SD: 2.7) | 0.313 |
| BMI | (kg/m^2^) | 20.8 (SD: 2.5) | 20.4 (SD: 2.1) | 0.243 |
| Alcohol consumption | More than 4 times per week | 5 (2.4%) | 2 (0.9%) | 0.406 |
|  | Less than once per month | 85 (41.1%) | 96 (44.7%) |  |
|  | Never | 117 (56.5%) | 117 (54.4%) |  |
| Use of supplement  containing vitamin D | No | 275 (94.5%) | 282 (96.6%) | 0.225 |
|  | Yes | 16 (5.5%) | 10 (3.4%) |  |
| Exercise habit | More than twice per week | 18 (6.2%) | 37 (12.7%) | 0.041 |
|  | Once per week | 45 (15.5%) | 48 (16.4%) |  |
|  | 1 to 2 times per month | 67 (23.0%) | 68 (23.3%) |  |
|  | Rarely | 161 (55.3%) | 139 (47.6%) |  |
| Suntan within the past 12 months | No | 143 (49.1%) | 158 (54.1%) | 0.230 |
|  | Yes | 148 (50.9%) | 134 (45.9%) |  |
| Sun exposure in the last 3 months | Always | 22 (7.6%) | 36 (12.3%) | 0.227 |
|  | More often than not | 65 (22.3%) | 52 (17.8%) |  |
|  | Sometimes | 46 (15.8%) | 52 (17.8%) |  |
|  | Infrequently | 58 (19.9%) | 51 (17.5%) |  |
|  | Never | 100 (34.4%) | 101 (34.6%) |  |
| Time regularly spent  outside on weekday | (min/d) | 40 (25-60) | 40 (25-65) | 0.658 |
| Time spent outside on weekdays | ≥3 h/d | 57 (19.6%) | 77 (26.4%) | 0.067 |
|  | 2 to <3 h/d | 179 (61.5%) | 147 (50.3%) |  |
|  | 1 to <2 h/d | 15 (5.2%) | 13 (4.5%) |  |
|  | <1 h/d | 11 (3.8%) | 17 (5.8%) |  |
|  | Rarely | 29 (10.0%) | 38 (13.0%) |  |
| Time regularly spent  outside on weekends | (min/d) | 25 (10-50) | 30 (10-62.5) | 0.076 |
| Time spent outside on weekends | ≥3 h/d | 49 (16.8%) | 60 (20.5%) | 0.107 |
|  | 2 to <3 h/d | 141 (48.5%) | 118 (40.4%) |  |
|  | 1 to <2 h/d | 20 (6.9%) | 30 (10.3%) |  |
|  | <1 h/d | 14 (4.8%) | 23 (7.9%) |  |
|  | rarely | 67 (23.0%) | 61 (20.9%) |  |
| Sunscreen use | Always | 77 (26.5%) | 103 (35.3%) | 0.140 |
|  | More often than not | 55 (18.9%) | 51 (17.5%) |  |
|  | Sometimes | 45 (15.5%) | 31 (10.6%) |  |
|  | Infrequently | 44 (15.1%) | 38 (13.0%) |  |
|  | Never | 70 (24.1%) | 69 (23.6%) |  |
| Sunscreen use for arms and legs | No | 122 (41.9%) | 123 (42.1%) | 0.961 |
|  | Yes | 169 (58.1%) | 169 (57.9%) |  |
| Paying attention to UV exposure | No | 114 (39.2%) | 110 (37.7%) | 0.709 |
|  | Yes | 177 (60.8%) | 182 (62.3%) |  |
| Recently wearing clothes | Arms and legs exposed | 173 (59.5%) | 176 (60.3%) | 0.839 |
|  | Without skin exposed | 118 (40.5%) | 116 (39.7%) |  |
| Skin type | V and VI: Dark brown  or black skin | 70 (24.1%) | 85 (29.1%) | 0.231 |
|  | IV: Light brown skin  (Burns minimally, tans easily) | 0 (0.0%) | 2 (0.7%) |  |
|  | III: Darker white skin  (Tans after initial burn) | 18 (6.2%) | 11 (3.8%) |  |
|  | II: Fair skin, blue eyes  (Burns easily, tans poorly) | 86 (29.6%) | 93 (31.8%) |  |
|  | I: Pale white skin,  blue/green eyes,  blond/red hair  (Always burns, does not tan) | 21 (7.2%) | 21 (7.2%) |  |
| Habitual intake of fish | Less than 1 day a week | 74 (25.4%) | 72 (24.7%) | 0.610 |
|  | 1 to 2 days a week | 104 (35.7%) | 110 (37.7%) |  |
|  | 2 to 3 days a week | 106 (36.4%) | 98 (33.6)% |  |
|  | More than 4 days a week | 7 (2.4%) | 12 (4.1%) |  |
| Habitual intake of the vitamin D | Less than 1 day a week | 167 (57.4%) | 163 (55.8%) | 0.789 |
| abundant fish (e.g. salmon, | 1 to 2 days a week | 89 (30.6%) | 95 (32.5%) |  |
| sardine, saury, flounder, eel, | 2 to 3 days a week | 33 (11.3%) | 30 (10.3%) |  |
| herring and grunt) | More than 4 days a week | 2 (0.7%) | 4 (1.4%) |  |
| Frequency of low-fat milk intake | More than once a week | 35 (12.1%) | 39 (13.4%) | 0.641 |
|  | Less than once a week | 255 (87.9%) | 253 (86.6%) |  |
| Frequency of milk intake | More than once a week | 119 (40.9%) | 124 (42.5%) | 0.700 |
|  | Less than once a week | 172 (59.1%) | 168 (57.5%) |  |
| Frequency of yogurt intake | More than once a week | 160 (55.4%) | 178 (61.0%) | 0.172 |
|  | Less than once a week | 119 (40.9%) | 124 (42.5%) | 0.700 |
| Frequency of cheese intake | More than once a week | 112 (38.5%) | 126 (43.2%) | 0.252 |
|  | Less than once a week | 179 (61.5%) | 166 (56.8%) |  |
| Frequency of cottage cheese intake | More than once a week | 5 (1.7%) | 9 (3.1%) | 0.282 |
|  | Less than once a week | 286 (98.3%) | 283 (96.9%) |  |
| Frequency of milk beverage drink | More than once a week | 34 (11.7%) | 40 (13.7%) | 0.465 |
|  | Less than once a week | 257 (88.3%) | 252 (86.3%) |  |
| Frequency of butter intake | More than once a week | 66 (22.7%) | 77 (26.4%) | 0.301 |
|  | Less than once a week | 225 (77.3%) | 215 (73.6%) |  |
| Frequency of margarine intake | More than once a week | 43 (14.8%) | 44 (15.1%) | 0.921 |
|  | Less than once a week | 248 (85.2%) | 248 (84.9%) |  |
| Frequency of dried fish intake | More than once a week | 23 (7.9%) | 22 (7.5%) | 0.867 |
|  | Less than once a week | 268 (92.1%) | 270 (92.5%) |  |
| Frequency of fish eaten with the bone intake | More than once a week | 25 (8.6%) | 26 (8.9%) | 0.894 |
|  | Less than once a week | 266 (91.4%) | 266 (91.1%) |  |
| Frequency of tuna intake | More than once a week | 33 (11.3%) | 34 (11.6%) | 0.909 |
|  | Less than once a week | 258 (88.7%) | 258 (88.4%) |  |
| Frequency of eel intake | More than once a week | 1 (0.3%) | 1 (0.3%) | 0.998 |
|  | Less than once a week | 290 (99.7%) | 291 (99.7%) |  |
| Frequency of white fish intake | More than once a week | 55 (18.9%) | 70 (24.0%) | 0.136 |
|  | Less than once a week | 236 (81.1%) | 222 (76.0%) |  |
| Frequency of blueback fish intake | More than once a week | 73 (25.1%) | 83 (28.4%) | 0.363 |
|  | Less than once a week | 218 (74.9%) | 209 (71.6%) |  |
| Frequency of red fish intake | More than once a week | 88 (30.2%) | 93 (32.0%) | 0.654 |
|  | Less than once a week | 203 (69.8%) | 198 (68.0%) |  |
| Frequency of egg | More than once a week | 270 (92.8%) | 273 (93.5%) | 0.735 |
|  | Less than once a week | 21 (7.2%) | 19 (6.5%) |  |
| Frequency of mushrooms | More than once a week | 205 (70.4%) | 204 (69.9%) | 0.878 |
|  | Less than once a week | 86 (29.6%) | 88 (30.1%) |  |

The frequency of intake of each food item was expressed as eight categories (at least twice a day, once a day, 4-6 times a week, 2-3 times a week, once a week, 2-3 times a month, once a month, and not at all) and was reclassified into two categories: more than once a week and less than once a week.

Continuous variables are expressed as mean (standard deviation) or median (Q1-Q3), and categorical variables are expressed as frequency (percentage) using the Wilcoxon rank-sum test for continuous variables and the chi-squared test for categorical variables.

SD: standard deviation, 25(OH)D: 25-hydroxyvitamin D, 24,25(OH)_2_D_3_: 24,25-dihydroxycholecalciferol, iPTH: intact parathyroid hormone, UV-B: ultraviolet-B, BMI: body mass index.

**Supplementary Table 2.** Association between DHQ-calculated energy and nutrition intakes and VDD in the development set

| **Variable** | **25(OH)D <20** | **25(OH)D >=20** | **p-value** |
| --- | --- | --- | --- |
|  | **n=199** | **n=92** |  |
| Energy (kJ) (crude) | 6,547 (SD: 1,694) | 6,834 (SD: 1,987) | 0.147 |
| Protein (g) (crude) | 54.5 (SD: 16.5) | 60.4 (SD: 20.2) | 0.019 |
| Fat (g) (crude) | 57.8 (SD: 22.3) | 59.5 (SD: 24.8) | 0.537 |
| Carbohydrates (g) (crude) | 199.4 (SD: 47.7) | 208.1 (SD: 56.7) | 0.144 |
| Calcium (mg)(crude) | 418 (SD: 195) | 484 (SD: 223) | 0.015 |
| Vitamin D (µg)(crude) | 4.5 (SD: 2.4) | 5.9 (SD: 3.4) | 0.001 |
| Mushroom intake (g) (crude) | 11 (SD: 11) | 14 (SD: 15) | 0.628 |
| Fish intake (g) (crude) | 36 (SD: 21) | 44 (SD: 30) | 0.042 |
| Egg intake (g) (crude) | 38 (SD: 32) | 43 (SD: 38) | 0.236 |
| Milk intake (g) (crude) | 96 (SD: 118) | 133 (SD: 130) | 0.004 |
| Protein (g/4,184 kJ) (energy adjusted) | 34.7 (SD: 5.1) | 36.9 (SD: 5.89) | 0.008 |
| Fat (g/4,184 kJ) (energy adjusted) | 36.3 (SD: 6.5) | 35.7 (SD: 6.6) | 0.247 |
| Carbohydrates (g/4,184 kJ) (energy adjusted) | 129.0 (SD: 16.9) | 129.0 (SD: 16.9) | 0.399 |
| Calcium (mg/4,184 kJ) (energy adjusted) | 264 (SD: 96) | 292 (SD: 93) | 0.004 |
| Vitamin D (µg/4,184 kJ) (energy adjusted) | 2.8 (SD: 1.3) | 3.5 (SD: 1.7) | <0.0001 |
| Mushroom intake (g/4,184 kJ) (energy adjusted) | 7 (SD: 7) | 8 (SD: 8) | 0.712 |
| Fish intake (g/4,184 kJ) (energy adjusted) | 22 (SD: 12) | 27 (SD: 16) | 0.025 |
| Egg intake (g/4,184 kJ) (energy adjusted) | 60 (SD: 70) | 80 (SD: 74) | 0.005 |
| Milk intake (g/4,184 kJ) (energy adjusted) | 24 (SD: 17) | 26 (SD: 21) | 0.528 |

Continuous variables are expressed as mean (standard deviation) or median (Q1-Q3), and categorical variables are expressed as frequency (percentage) using the Wilcoxon rank-sum test for continuous variables.

VDD: vitamin D deficiency

**Supplementary Table 3**. Alternative two multivariable models for VDD in the development set.

| **Variable [reference]**  **Multivariable regression model** | **Category** | **Multivariable regression model (n=291)** | | | **C-index** |
| --- | --- | --- | --- | --- | --- |
|  |  | **Odds ratio** | **95% confidence interval** | **p-value** |  |
| **Model 2 (excludes season)** |  |  |  |  | 0.750 |
| The residential area（ref=Kanto region） | Kyushu/Okinawa | 3.77 | 1.84-7.71 | <.001 |  |
|  | Chubu/Kinki/Shikoku | 3.09 | 1.43-6.67 | 0.004 |  |
|  | Hokkaido/Tohoku | 2.82 | 1.24-6.43 | 0.014 |  |
| BMI category [≥18.5 kg/m^2^] | <18.5 kg/m^2^ | 3.02 | 1.15-7.93 | 0.025 |  |
| Use of supplement containing vitamin D (ref=Yes) | No | 9.01 | 2.81-28.86 | <.001 |  |
| Time regularly spent outside on weekdays (ref= ≥60 min) | <60 min | 1.64 | 0.90-2.97 | 0.104 |  |
| Frequency of vitamin D abundant fish intake (ref=≥once a week) | <once a week | 2.24 | 1.28-3.92 | 0.005 |  |
| Frequency of dried fish intake (ref=≥once a week) | <once a week | 1.42 | 0.57-3.57 | 0.453 |  |
| **Model 3 (excludes season and added the UV-B irradiation)** |  |  |  |  | 0.755 |
| The residential area（ref=Kanto region） | Kyushu/Okinawa | 3.79 | 1.84-7.80 | <.001 |  |
|  | Chubu/Kinki/Shikoku | 3.32 | 1.52-7.26 | 0.003 |  |
|  | Hokkaido/Tohoku | 2.64 | 1.15-6.06 | 0.022 |  |
| BMI category [≥18.5 kg/m^2^] | <18.5 kg/m^2^ | 2.84 | 1.09-7.41 | 0.033 |  |
| Use of supplement containing vitamin D (ref=Yes) | No | 9.46 | 2.95-30.39 | <.001 |  |
| Time regularly spent outside on weekdays (ref= ≥60 min) | <60 min | 1.57 | 0.86-2.87 | 0.140 |  |
| Frequency of vitamin D abundant fish intake (ref=≥once a week) | <once a week | 2.31 | 1.32-4.06 | 0.004 |  |
| Frequency of dried fish intake (ref=≥once a week) | <once a week | 1.34 | 0.53-3.35 | 0.538 |  |
| The UV-B irradiation (100 mJ/cm^2^) 30-day |  | 0.92 | 0.83-1.01 | 0.070 |  |

VDD: vitamin D deficiency, BMI: body mass index
