# Supplementary material for: Development of a predictive scoring system for vitamin D deficiency ‘Vitamin D Deficiency Predicting Scoring (ViDDPreS)’ based on the vitamin D status in young Japanese women: a nationwide cross-sectional study
Source: Public Health Nutr. 2024 Sep 27;27(1):e185. doi: 10.1017/S1368980024001708 (PMC11505081; doi:10.1017/S1368980024001708)
Supplement: Kuwabara et al. supplementary material 2 — Kuwabara et al. supplementary material [file S1368980024001708sup002.docx]

**Detailed methods**

1. ***Serum 25(OH)D concentration-related variables as outcomes***

Serum vitamin D metabolites, 25(OH)D_2_, 25(OH)D_3_ and 24,25-dihydroxycholecalciferol (24,25(OH)_2_D_3_), were measured using modified method of liquid chromatography- tandem mass spectrometry (LC-APCI-MS/MS) ^(1)^. The modification point was derivation of extracted vitamin D metabolites with 4-[2-(6,7-dimethoxy-4-methyl-3-oxo-3,4-dihydroquinoxalyl) ethyl]-1,2,4-triazoline-3,5-dione (DMEQ-TAD) to obtain high sensitivity by increasing ionization efficiency ^(2)^. Their summation was used to calculate the total serum 25(OH)D levels.

All the serum samples were collected from a single laboratory. SRM 972a (The National Institute of Standards and Technology, NIST) which consists of four vials of level 1-4 frozen serum from healthy donors with different 25(OH)D concentrations, was used to estimate the accuracy. The concentrations of 25(OH)D_2_ and 25(OH)D_3_ in SRM 972a measured by our LC-APCI-MS/MS were 0.8±1.0% and 0.9±1.9% to the assigned values, respectively. The results satisfied the criteria for the LC-MS/MS performance threshold of the AOAC procedure for 25OH vitamin D standardization/validation (VDSP) ^(3)^. The coefficients of variation (CV) of intra- and inter-assay for Total 25(OH)D level in this study were 1.5- 8.3% and 4.6%.

References

1. Tsugawa N, Suhara Y, Kamao M et al (2005) Determination of 25-hydroxyvitamin D in human plasma using high-performance liquid chromatography--tandem mass spectrometry. *Anal Chem* **77**, 3001-3007.
2. Higashi T, Awada D, Shimada K (2001) Simultaneous determination of 25-hydroxyvitamin D2 and 25-hydroxyvitamin D3 in human plasma by liquid chromatography-tandem mass spectrometry employing derivatization with a Cookson-type reagent. *Biol Pharm Bull* **24**, 738-743.
3. Ihara H, Kiuchi S, Ishige T et al (2018) Surveillance evaluation of the standardization of assay values for serum total 25-hydroxyvitamin D concentration in Japan. *Ann Clin Biochem* **55**, 647-656.
4. ***Estimation of the cumulative ambient UV-B irradiation***

The ultraviolet-B (UV-B) radiation flux density was calculated using a radiative-transfer code called ‘Simple Model of the Atmospheric Radiative Transfer of Sunshine, version 2 (SMARTS2)’ developed by Gueymard to calculate the flux density of solar radiation on the ground surface E(λ) for wavelengths between 280 and 4,000 nm ^(1)^. Based on this, we estimated the cumulative ambient UV-B irradiation from 1 month to 1 d before the blood was drawn.

In particular, for wavelengths from 280 to 1,700 nm, calculations can be made at 1 nm intervals. UV spectrum of wavelength *λ* at the horizontal plane surface is expressed as follows;

$$E\left( \lambda\right)=E_{bn}\left( \lambda\right)\cos\theta_{z}+I_{d}(\lambda)$$

where

*E* (*λ*): UV flux density of the wavelength, *λ*,

*E_bn_* (*λ*): UV spectrum of the direct component of solar radiation,

*θ_z_*: solar zenith angle,

*I_d_* (*λ*): UV spectrum of the diffusive component of solar radiation.

This equation allows us to calculate the UV spectrum Ebn (λ) and Id (λ) at the ground surface for a given season, time, and location under a cloudless sky. We applied this calculation to the sites where the subjects were recruited at each participating university or college. The UV flux reaching the top of the atmosphere from the sun was corrected for the distance between the earth and sun for each day of the year. The total ozone at each site is given by measurements from NASA’s OM ^(2)^ and OMPS ^(3)^ satellites that regularly report ozone total column data. The optical thickness of aerosols was observed under a cloudless sky at the Tsukuba station, Japan Meteorological Agency, using a sun-photometer that split the direct solar radiation into 368, 500, 675, 778, and 862 nm, yielding the Ångström parameters α and β. The aerosol data for Tsukuba were used when there were no data available for aerosols at the study sites. Rayleigh scattering is related to the number of air molecules that can generate atmospheric pressure. UV spectra have been observed by the Japan Meteorological Agency, every hour daily with Brewer spectra available for every 0.5 nm interval ^(4)^.

References

1. Florida Solar Energy Center (1995) Report FSEC-PF-270-95. http://www.fsec.ucf.edu/en/publications/pdf/FSEC-PF-270-95.pdf (accessed by May 2023)
2. Liu X, Bhartia PK, Chance K, Spurr RJD, Kurosu TP. Ozone profile retrievals from the Ozone Monitoring Instrument. Atmos. Chem. Phys. 2010;10, 2521-2537. https://doi.org/10.5194/acp-10-2521-2010.]
3. Flynn L, Long C, Wu X, Evans R, Beck CT, Petropavlovskikh I, et al. Performance of the Ozone Mapping and Profiler Suite (OMPS) products. J Geophys Res Atmos. 2014;199: 6181-6195, doi:10.1002/2013JD020467)
4. Japan Meteorological Agency. Ozone Layer and Ultraviolet Radiation; 2021. Available from: https://www.data.jma.go.jp/gmd/env/ozonehp/ozone_daily.html]
